# An intraepithelial ILC1-like natural killer cell subset produces IL-13

**DOI:** 10.3389/fimmu.2025.1521086

**Published:** 2025-03-06

**Authors:** Sainiteesh Maddineni, Krishna Sharma, Imran A. Mohammad, Alistaire D. Ruggiero-Sherman, Ivan Stepanek, June Ho Shin, Jennifer K. Bando, John B. Sunwoo

**Affiliations:** ^1^ Department of Otolaryngology — Head & Neck Surgery, Stanford University School of Medicine, Palo Alto, CA, United States; ^2^ Department of Microbiology & Immunology, Stanford University School of Medicine, Palo Alto, CA, United States

**Keywords:** NK cells, tumor immunology, IL-13, cytokines, ILC, allergy, HNSCC

## Abstract

Natural killer (NK) cells are innate immune effectors with considerable heterogeneity and potent antitumor capabilities. Intraepithelial ILC1 (ieILC1)-like NK cells, a population of cytotoxic tissue-resident innate lymphoid cells, have recently been documented in the microenvironment of head and neck squamous cell carcinomas (HNSCC) and other solid tumors. These cells have antitumor cytolytic potential and are potent producers of type 1 cytokines, including IFNγ. Here, we identify a subpopulation of ex vivo differentiated ieILC1-like NK cells that produce IL-13 upon stimulation. Functional characterization revealed that these cells co-expressed IFNγ and IL-13 while maintaining an ILC1 transcriptional signature. IL-13 was induced either upon co-culture with tumor cell lines, or in response to TGF-β and IL-15. IL-13-expressing ieILC1-like NK cells were identified among tumor infiltrating lymphocytes expanded from patient HNSCC tumors, in support of their *in vivo*existence in primary tumors. These data demonstrate additional heterogeneity within the ieILC1-like NK cell population than previously appreciated and highlight a unique form of ILC plasticity in which cells with clear ILC1 transcriptional profiles express a type 2 cytokine. With the known roles of IL-13 in cancer cell growth dynamics and immunoregulation, the identification of this subset within tumor microenvironments presents a potential target for therapeutic manipulation.

## 1 Introduction

The immune response to cancer is broad and involves numerous immune subtypes. Traditionally, cytotoxic T cells have been considered the main effectors of antitumor immunity (1). However, other immune effectors can contribute significantly to the immune response. Natural killer (NK) cells are innate lymphoid cells (ILCs) with cytotoxic functions and include subsets that parallel those of T cells (2). NK cells are a promising avenue for immunotherapy, as they offer potent cytotoxicity while bypassing the need for strict HLA matching and reduce the risk of graft-versus-host disease (GvHD), opening an avenue for allogeneic, “off-the-shelf” therapy (3–5). However, tumors present various factors, including hypoxia, TGF-β, and PGE2 that can suppress immune effector functions, including NK cytotoxicity (6). NK cells have traditionally been characterized as CD3^–^CD56^+^ immune cells found in the peripheral blood. More recently, tissue-resident NK (trNK) cells, expressing markers of tissue-residency like CD103, have been investigated and have been found to be quite heterogeneous between various tissue compartments, such as the liver, thymus, mucosa, and lymph nodes (2, 5, 7).

The ILC family is broadly categorized by the expression of critical transcription factors and the type of cytokines they produce (8). NK cells and ILC1s belong to the innate lymphoid cell type 1 (Group I ILC) family, which is characterized by the expression of T-bet and type 1 cytokines IFNγ and TNF. Group 2 ILCs, which include ILC2s, express GATA3 and the type 2 cytokines IL-13, IL-5, and IL-9. Finally, Group 3 ILCs, which include ILC3s, express Rorγt and the Th17-type cytokines IL-17 and IL-22 (8). Lymphoid Tissue inducer (LTi) cells are an additional ILC family member important for secondary lymphoid tissue development in embryogenesis (9). These cells also express Rorγt and secrete IL-17 and IL-22 like ILC3s (10). In addition to these three ILC classes, NK cells differ from ILC1s in that they express cytolytic granules with perforin and granzyme (8). In different tissues, NK cells can be differentiated from ILC1s by the expression of Eomes, the lack of CD127, and the absence of the Hobit. In the context of cancer, ILC1s may play a role in immunosurveillance, driven by IL-15 (11).

ILC2s have a distinct phenotype relative to ILC1s. ILC2s are associated with allergic responses and helminth infection, as well as tissue homeostasis and wound repair (12). ILC2s have canonically been implicated in the pathophysiology of allergic airway processes, including asthma and chronic rhinosinusitis (13). More recently, ILC2s have been associated with the pathophysiology of cancer; however, their roles remain conflicted. In hepatocellular carcinoma, ILC2s are reported to produce IL-13, CXCL2, and CXCL8 to recruit neutrophils producing arginase 1, which can suppress antitumor T cell responses (12). Additionally, PD-1^+^ ILC2s have been documented to enhance tumor growth, inhibiting NK cell responses and enhancing regulatory T cell (Treg) and myeloid-derived suppressor cell (MDSC) activity. These PD-1^+^ ILC2s have limited intra-tumoral accumulation and proliferation (14). These results suggest that ILC2s can have a potent pro-tumorigenic role in cancer. However, evidence supporting ILC2s as antitumor effectors have been noted. In melanoma and prostate cancer, ILC2s can activate CD103^+^ dendritic cells to prime CD8^+^ T cells (12). ILC2s can also secrete GM-CSF to recruit eosinophils (12). Recently, ILC2s have been observed to secrete granzyme B and lyse tumor cells via interaction of ILC2 DNAM-1 with tumor cell CD112 or CD155 (15).

The role of type 2 immune responses in cancer remains unclear. IL-13 is a key effector cytokine of ILC2s and Th2 CD4^+^ T cells with various physiological functions. In cancer, IL-13 can regulate tumor cell growth and influence immunosurveillance, with unclear data on whether IL-13 promotes or inhibits tumor progression (16). One of the IL-13 receptors, IL-13Rα2, has been found to have pro-tumorigenic signaling. IL-13Rα2 signaling can promote cancer survival, proliferation, invasion, and metastasis (17–19). Given these findings, IL-13Rα2-targeting antibodies and chimeric antigen receptors (CARs) are in therapeutic development for various cancers (19).

ILCs have been reported to have considerable plasticity. ILC2s and ILC3s have been reported to adopt an ILC1 phenotype via factors like IL-12 (11). Differentiation of ILC2s and ILC3s are thought to potentially explain elevations in ILC1s during inflammatory processes in chronic inflammatory diseases (11). ILC2 and ILC3-derived ILC1s can revert to ILC2s or ILC3s as well, under regulation of IL-4-producing eosinophils and IL-23-producing dendritic cells (11). Generally, NK cells do not have overlap of type 1 and type 2 cytokine profiles. An analysis of NK cell populations identified distinct IL-13^+^ and IFNγ^+^ NK cell subpopulations; IL-4 can induce IL-13^+^ NK cells, while IL-12 can induce IFNγ^+^ NK cells (20). IL-13^+^ NK cells were typically CD161^+^CD56^–^ and associated with TNFa and GM-CSF secretion as well. The co-expression of IL-13 and TNFa can suggest overlap of type 1 and type 2 cytokine profiles, but this was not intensely profiled (20). IFNγ^+^ NK cells are possibly a more mature phenotype, with IL-13^+^ NK cells representing an immature phenotype, with IL-13^+^IFNγ^+^ NK cells representing an intermediate state (21). Ultimately, IL-13^+^IFNγ^+^ NK cells appear to be a rare subpopulation primarily characterized *in vitro*.

In recent years, trNKs have been documented to have important roles in cancer. A novel subset of ILC1s, known as intraepithelial ILC1s (ieILC1s), is a tissue-resident population of ILC1s characterized by TGF-β imprinting and IFNγ production upon IL-15 stimulation (22). These cells were characterized as NKp44^+^CD103^+^ cells with T-bet and EOMES expression that expanded significantly in Crohn’s disease (22). Recently, ieILC1s were a distinct population identified in the microenvironment of primary HNSCC samples (23). These ieILC1s possess high *in vivo* antitumor potential and are characterized as CD49a^+^CD103^+^ cells. Peripheral NK cells can differentiate into ieILC1-like NK cells via co-culture with HNSCC tumor cells and IL-15, and the *ex vivo* differentiated ieILC1-like NK cells are robust producers of IFNγ and CD107a upon stimulation (23). In contrast, peripheral NK cells co-cultured with TGF-β and IL-15 have also been shown to differentiate into CD49a^+^CD103^+^ NK cells that express inhibitory markers like GITR and CD101 and suppress CD4^+^ T cells (24). Thus, the population of CD49a^+^CD103^+^ NK cells is heterogeneous and requires further investigation.

Here, we demonstrate a subset of ieILC1-like NK cells with IFNγ and IL-13 co-expression. These ieILC1-like NK cells retain all canonical features of ieILC1s while expressing this type 2 cytokine upon stimulation. Moreover, these IL-13^+^ ieILC1-like NK cells could be identified within the microenvironment of primary HNSCC samples, revealing them as a novel subpopulation of NK cells with potential clinical implications that require further investigation.

## 2 Methods

### 2.1 Tumor cell lines and culture

PCI-13 was obtained from Dr. Theresa Whiteside at the University of Pittsburgh. UM-SCC-47 was obtained from Dr. J. Chad Brenner at the University of Michigan. The SK-MEL-28 human melanoma cell line was obtained from the American Type Culture Collection. Tumor cells were grown in Dulbecco’s modified Eagle’s medium/F-12 50/50 supplemented with 10% heat-inactivated fetal bovine serum (FBS), 1% Pen Strep, and 1% non-essential amino acids. Tumor cells were harvested using TrypLE Express reagent (Gibco).

### 2.2 Peripheral blood NK cell isolation

De-identified human leukoreduction system chambers (LRS) were obtained from the Stanford Blood Center. NK cells from each donor sample were enriched by negative selection (RosetteSep Human NK Cell Enrichment Cocktail, Stem Cell Technologies), and then subjected to density gradient centrifugation at room temperature (Ficoll-Paque Premium, Cytvia). The PBMC layer enriched for NK cells was collected and washed with phosphate-buffered saline (PBS). Remaining red blood cells were lysed with ACK lysing buffer. Isolated NK cells were rested for 48 hours in RPMI 1640 supplemented with 10% FBS, 1% Pen Strep, 1% non-essential amino acids, 1mM sodium pyruvate, 10mM HEPES, 55µM 2-mercaptoethanol, and 10 ng/mL of IL-15 before experiments.

### 2.3 Peripheral blood NK cell differentiation

PCI-13 cells were harvested and irradiated at 100 Gy prior to co-culture. 1x10^6^ rested NK cells and 5x10^5^ irradiated PCI-13 cells were co-cultured in a 24-well plate in AIM V media with 5% Immune Cell SR and 10 ng/mL of IL-15. Media was replaced every 2 days, and fresh irradiated PCI-13 cells were added at day 4 in addition to the original tumor cells. At day 8, ieILC1-like NK cells were collected for subsequent assays. Co-cultures of NK cells with UM-SCC-47, SK-MEL-28, and K562 cell lines were similarly performed. In separate experiments, NK cells were cultured with 20 ng/mL of TGF-β in addition to IL-15.

### 2.4 xCelligence assay

Cytotoxicity was measured with the xCELLigence Real-time Cellular Analysis (Agilent), which uses electrical impedance to measure cell index. 2x10^4^ PCI-13 target cells were initially loaded and allowed to grow for 8 hours. 5x10^3^ ieILC1-like NK cells were then loaded in media containing 10 ng/mL of IL-15. Cytolysis was measured for 48 hours.

### 2.5 *In vitro* stimulation of NK cells

NK cells were stimulated for 6 hours with PMA/Ionomycin (50 ng/mL PMA and 1 µg/mL Ionomycin) or K562 cells, depending on assay. K562 cells used for stimulation were not irradiated and added at a 1:1 E:T ratio with NK cells. IL-25 was added at 100 ng/mL.

### 2.6 Antibody staining and flow cytometry analysis

Cells were stained with a viability dye (eBioscience Fixable Viability Dye eFluor 780), and then incubated with antibodies to extracellular antigens for 30 minutes at 4°C: CD3 APC/Cy7 (BioLegend Clone UCHT1), CD3 PerCP-Cy5.5 (BioLegend Clone OKT3), CD14 APC/Cy7 (BioLegend Clone HCD14), CD19 APC/Cy7 (BioLegend Clone HIB19), CD20 APC/Cy7 (BioLegend Clone 2H7), CD45 BV605 (BioLegend Clone 2D1), CD56 APC (BioLegend Clone HCD56), CD56 Pacific Blue (BioLegend Clone MEM-188), CD56 BUV737 (BD Horizon Clone NCAM16.2), CD49a BUV395 (BD OptiBuild Clone SR84), CD103 FITC (BioLegend Clone Ber-ACT8), IL-13Ra1 APC (SS12B), IL-13Ra2 APC (A21071B). In experiments with TIL cultures, True-Stain Monocyte Blocker (BioLegend) was used prior to staining to block non-specific antibody binding to monocytes/macrophages in culture.

In experiments involving intracellular staining, stained cells were fixed (BD Cytofix/Cytoperm) for 30 minutes at 4°C. Fixed cells were permeabilized (eBioscience Permeabilization Buffer) and stained for intracellular antigens for 1 hour at 4°C with the following antibodies: IFNγ Pacific Blue (BioLegend Clone B27), Ki67 BV421 (BioLegend Clone 11F6), CD107a BV711(BioLegend Clone H4A3), T-Bet PE/Cy7 (BioLegend Clone 4B10), GATA3 BV421(BioLegend Clone 16E10A23), Eomes BV421 (BD Horizon Clone X4-83), c-KIT PE (BioLegend Clone 104D2), CD161 PE (BioLegend Clone HP-3G10), CD127 PE (BioLegend Clone A019D5), IL-13Ra1 APC (BioLegend Clone SS12B), IL-13Ra2 APC (BioLegend Clone SHM38), IL-13 PE (BioLegend Clone JES10-5A2), and IL-13 APC (BioLegend Clone JES10-5A2). Stained cells were analyzed with a BD FACSymphony A5 flow cytometer, and fcs files were analyzed using FlowJo v10.10.0.

### 2.7 Luminex

Following *in vitro* stimulation, NK cells were collected in Eppendorf tubes and centrifuged at 300g for 5 minutes to pellet cells. Supernatants from each tube were collected in separate Eppendorf tubes and centrifuged again for 1000g for 5 minutes to remove additional debris. Supernatants were collected and stored at -80°C. Samples were analyzed by the Human Immune Monitoring Center at Stanford via a 48-plex Luminex Immuno-Assay for human cytokines. All samples were measured in duplicate.

### 2.8 qRT-PCR

RNA was extracted from isolated NK cells using a QIAGEN RNeasy Plus Mini kit. RNA concentrations were normalized, and cDNA synthesis was completed with the Vazyme HiScript III All-in-one RT SuperMix Perfect for qPCR kit. qPCR of cDNA was then performed using the Taqman Gene Expression Assay. Taqman probes (Thermo Fisher Scientific) were selected based on the recommended best coverage probe for each analyzed gene: *IL13*. *GAPDH* was used as a reference gene; fold change in gene expression was measured relative to *GAPDH* expression for each sample. Fold change was calculated using the delta-delta Ct calculation method.

### 2.9 IL-13 Sorting and RNA library preparation

IL-13-expressing cells were identified using a IL-13 capture assay (Miltenyi Biotec IL-13 Secretion Assay), in which secreted IL-13 was captured by a cell surface-bound antibody. Cells with IL-13 captured at the cell surface were then purified using a BD FACSAria II. Cells were sorted into RPMI 1640 supplemented with 20% FBS, 1% Pen Strep, 1% non-essential amino acids, 1mM sodium pyruvate, 10mM HEPES, 55µM 2-mercaptoethanol, and 10ng/mL of IL-15. RNA was extracted from purified cells via the QIAGEN RNeasy Plus Mini kit and then stored at -80°C.

### 2.10 RNA sequencing and analysis

RNA library construction and sequencing was completed by MedGenome. RNA library preparation was done with the Takara SMART-Seq mRNA kit or Illumina Stranded mRNA preparation. FASTQ files were processed via the nf-core/rnaseq bioinformatics pipeline (25). Briefly, sequences were aligned with the STAR method and quantified via RSEM. Differential gene expression analysis (DGEA) was completed with the DESeq2 package (26). DGEA data was further processed and visualized with the Independent Hypothesis Weighting, VSN, pheatmap, and EnhancedVolcano packages in R (27–30).

### 2.11 Expanded primary tumor infiltrating lymphocyte culture

Viable HNSCC samples were obtained from the Stanford Tissue Bank through a protocol approved by the Stanford Institutional Review Board. The samples were washed twice in PBS, and 1-3 mm^3^ pieces of tumor were cultured for TIL expansion in a 24 well tissue culture plate with RPMI 1640 media with 10% FBS, 1% Antibiotic-Antimycotic, 1% non-essential amino acids, 1mM sodium pyruvate, 10mM HEPES, 55µM 2-mercaptoethanol, 1% GlutaMAX, and 6000 U/mL of recombinant human IL-2. After 4-7 days of culture, TIL cultures were transferred to plates that had been pre-coated with OKT3 antibody either overnight at 4°C or for 2 hours at 37°C. Soluble anti-CD28 was added at 5mg/mL to each well. 50% of media was replaced every 2-3 days with IL-2 supplementation at 3000 U/mL. TILs were then removed from culture and separately stimulated with PMA/Ionomycin or K562 cells as described above.

### 2.12 Published single cell RNA sequencing (scRNAseq) dataset analysis

scRNAseq datasets were derived from existing published data by Cella et al. and Tang et al. (31, 32). Analysis was done in R with Seurat (33). For data from the Cella et al. manuscript, we contacted the corresponding author of the publication and acquired processed data to generate a Uniform Manifold Approximation and Projection (UMAP) feature plot of IL-13 expression. For data from Tang et al. cells with fewer than 200 features were filtered out. Gene expression was log normalized. Principal component analysis was run on variable features and cells were clustered and represented with Uniform Manifold Approximation and Projection (UMAP) feature plots with expression of IL-13.

### 2.13 The Cancer Genome Atlas survival analysis

TCGA analysis was done in R using the TCGAbiolinks, survminer, survival, and DESeq2 packages (26, 34–38). Data was acquired from the TCGA-HNSC project available in the GDC Data Portal. We stratified patients in the extracted cohort by median IL-13 expression. We generated Kaplan-Meier curves of 10-year overall survival with p-value calculated using the log-rank test to compare patients with higher than median IL-13 expression to those with below median expression.

### 2.14 Software and statistical analysis

DGEA for bulk RNA sequencing and scRNAseq analysis of published data was completed in R. Statistical tests and visualizations were run in GraphPad Prism 10. Flow cytometry analysis was performed using FlowJo v10.10.0. Unless otherwise specified, statistical analyses were conducted with the student’s t-test at an alpha value of 0.05.

## 3 Results

**Figure 1:**
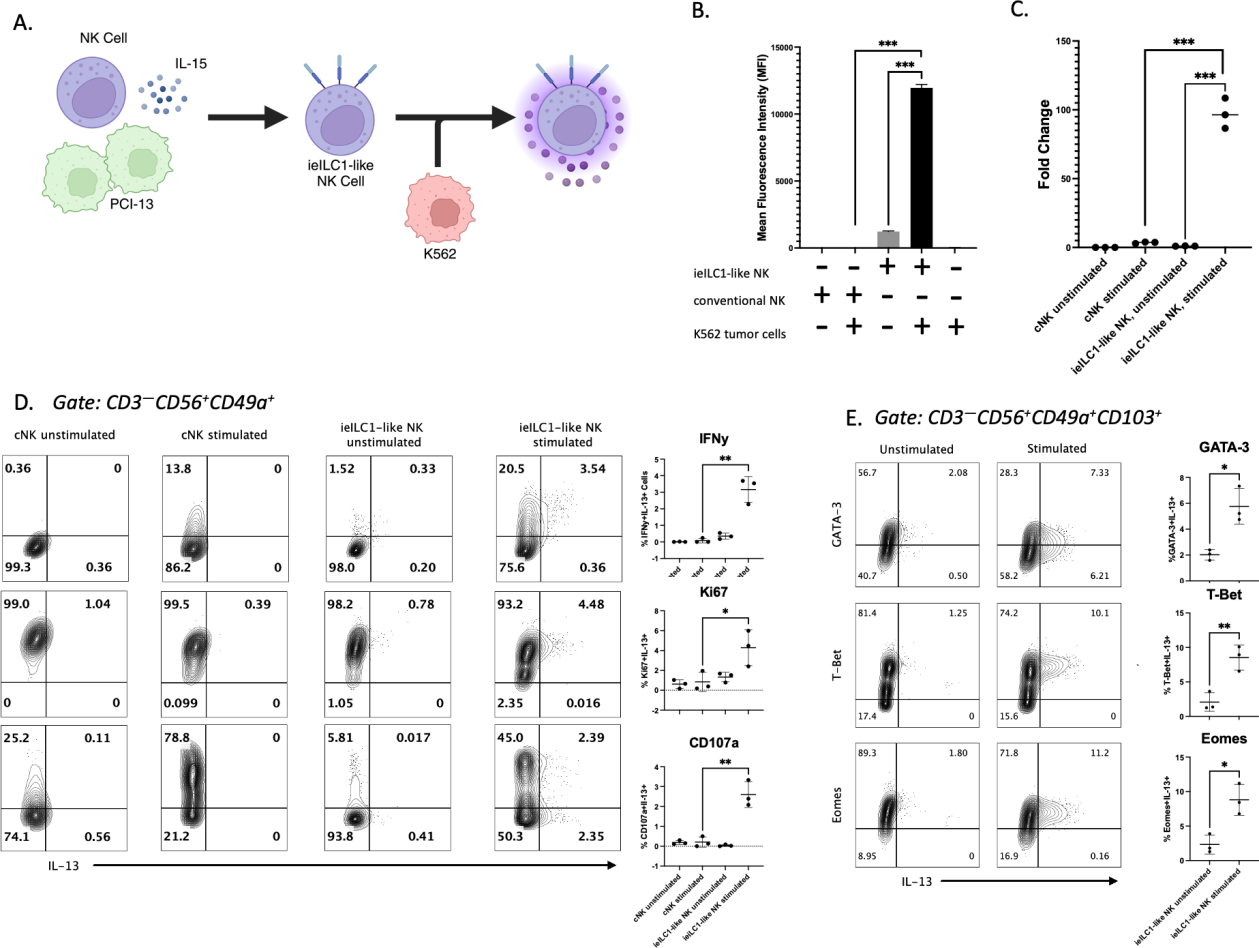
Identification of IL-13 producing ieILC1-like NK cells. **(A)** Schematic of ieILC1-like NK cell differentiation via co-culture of peripheral blood-derived NK cells with PCI-13 HNSCC and IL-15. **(B)** IL-13 levels measured in supernatant via Luminex bead immunoassay. Figure shows representative plot of n=3 donors analyzed. **(C)** *IL13* gene expression levels measured via qRT-PCR. Figure shows representative plot of n=3 donors analyzed. **(D)** Flow cytometry analysis of cytotoxicity and proliferation markers in stimulated CD49a^+^ NK cells. Figure shows representative plot of n=3 donors analyzed. Summary plots for all 3 donors are shown to the right. **(E)** Transcription factor analysis of IL-13^+^ ieILC1-like NK cells via flow cytometry. Figure shows representative plot of n=3 donors analyzed. Summary plots for all 3 donors are shown to the right. *p<0.05, **p<0.01, ***p<0.0001.

Our lab previously showed that co-culturing peripheral blood NK cells with HNSCC and IL-15 induced ieILC1-like NK cells, characterized as CD3^–^CD56^+^CD49a^+^CD103^+^ NK cells (23), (**Figure 1A**; **Supplementary Figure 1A**). These ieILC1-like NK cells showed potent cytolytic activity against PCI-13 HNSCC target cells (**Supplementary Figure 1B**).

To assess the cytokine profile of ieILC1-like NK cells, we performed a Luminex immunoassay to measure cytokines secreted after PMA/Ionomycin stimulation or K562 co-culture (**Supplementary Figure 1C**). ieILC1-like NK cells stimulated with K562 tumor cells secreted more IL-13 than unstimulated ieILC1-like NK cells (**Figure 1B**). In contrast, cNKs, which were NK cells from the same donor cultured in IL-15 alone, did not produce significant IL-13 in either stimulation condition. In support of these findings, we found that ieILC1-like NK cells stimulated with PMA/Ionomycin had higher expression of *IL13* compared to unstimulated ieILC1-like NK cells or cNKs as measured by quantitative reverse transcriptase PCR (qRT-PCR) (**Figure 1C**). Thus, we observed that IL-13 is produced by stimulated ieILC1-like NK cells.

Next, we assessed the frequency of ieILC1-like NK cells that produced IL-13 protein by flow cytometry. cNKs and ieILC1-like NK cells were obtained from the same donor and stimulated by co-culturing with K562 cells. Among the stimulated ieILC1-like NK cells, we observed an IFNγ^+^IL-13^–^ population and a smaller IFNγ^+^IL-13^+^ population (**Figure 1D**). No significant IFNγ^–^IL-13^+^ population was observed. cNKs did not express IL-13, consistent with the Luminex results. When we assessed surface CD107a expression as a proxy for degranulation, we observed a CD107a^–^IL-13^+^ population in addition to a CD107a^+^IL-13^+^ population. Finally, IL-13^+^ cells observed were Ki67^+^, an indication of their proliferative capability. Collectively, these data demonstrate that there is a subset of activated ieILC1-like NK cells capable of producing IL-13 upon tumor cell stimulation.

To confirm that IL-13^+^ ieILC1-like NK cells were members of the NK cell lineage and not contaminating ILC2s, we performed flow cytometry analysis of transcription factors T-bet, EOMES, and GATA3 (**Figure 1E**). IL-13^+^ ieILC1-like NK cells maintained high levels of T-bet and EOMES, consistent with the NK lineage, and expressed intermediate levels of GATA-3, consistent with all lymphocytes. Additionally, characterization of other ILC2-affiliated markers did not reveal robust differential expression of KLRB1 or IL-7R between IL-13^–^ and IL-13^+^ cells (**Supplementary Figure 1D**). cKIT expression appears to be slightly increased in IL-13+ cells, but this requires further investigation.

**Figure 2:**
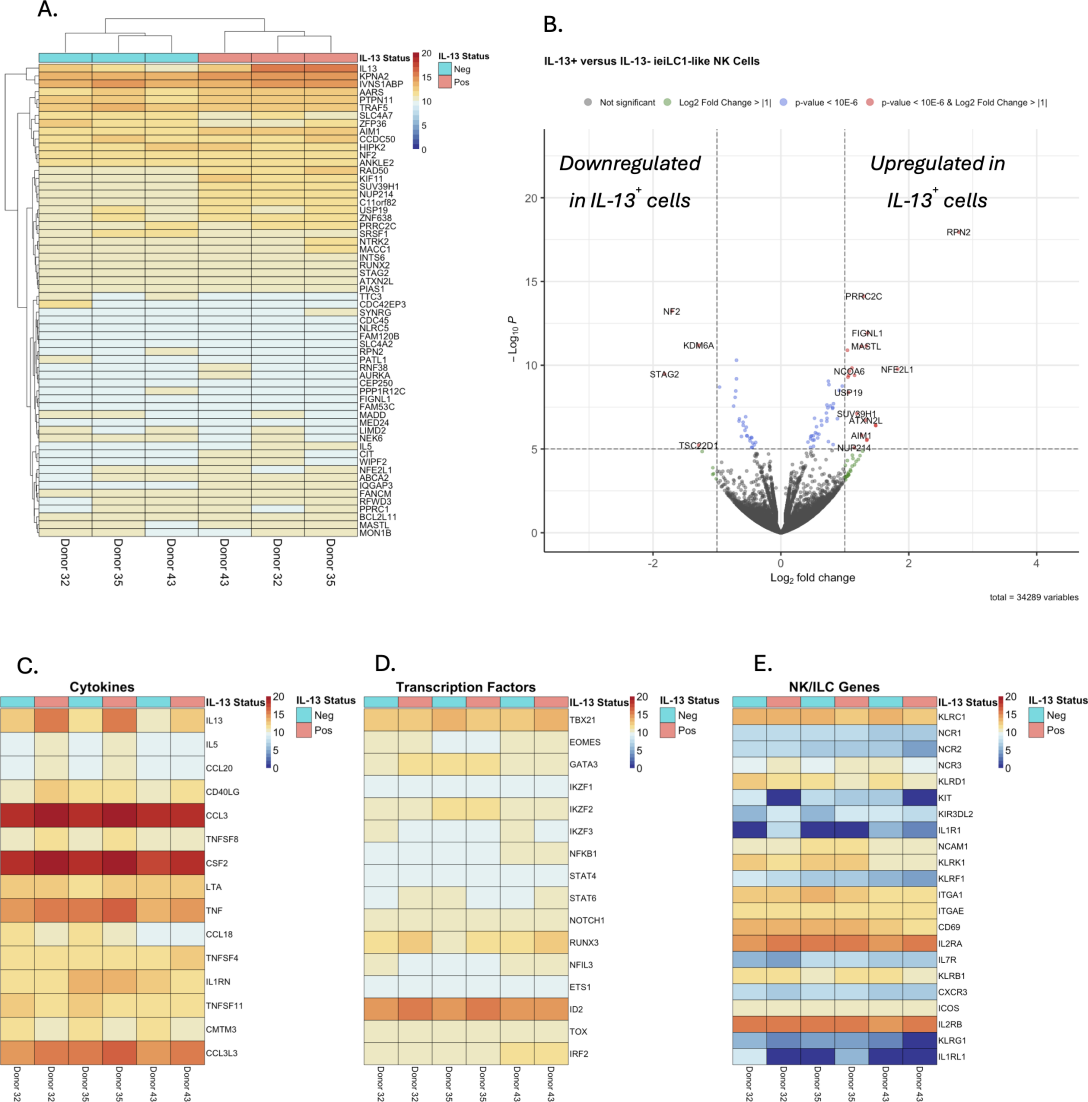
Differential gene expression analysis of bulk RNAseq data from IL-13^+^ and IL-13^–^ sorted, stimulated ieILC1-like NK cells. **(A)** Heatmap analysis of the top 60 differentially expressed genes sorted by false discovery rate. **(B)** Volcano plot of differential gene expressional analysis. **(C–E)** Heatmaps of selected gene sets for **(C)** cytokines, **(D)** transcription factors, and **(E)** NK/ILC associated genes.

To better understand the unique properties of IL-13^+^ieILC1-like NK cells compared to IL-13^–^ ieILC1-like NK cells, we profiled the cells by bulk RNA sequencing (RNA-seq). Following stimulation of ieILC1-like NK cells by PMA/ionomycin, we sorted IL-13^+^ and IL-13^–^ cells via a cytokine secretion capture assay and flow cytometry (**Supplementary Figure 2A**). Increased *IL13* expression was confirmed by qRT-PCR of the sorted IL-13^+^ cells (**Supplementary Figure 2B**). Differential gene expression analysis (DGEA) using the RNA-seq data (**Figures 2A, B**) also confirmed increased *IL13* expression in the sorted IL-13^+^ group (**Supplementary Figure 2C**). Heatmap analysis of cytokines showed that *IL5* was significantly expressed in addition to *IL13* (**Figure 2C**). Moreover, *CCL20* was highly expressed in only the IL-13^+^ group. Analysis of selected transcription factors showed no significant difference in expression of *TBX21* (T-bet), *EOMES*, and *GATA3* between the IL-13^+^ and IL-13^–^ groups (**Figure 2D**). An analysis of NK cell and ILC-associated genes did not identify any differentially expressed genes between IL-13^–^ and IL-13^+^ cells (**Figure 2E**).

**Figure 3:**
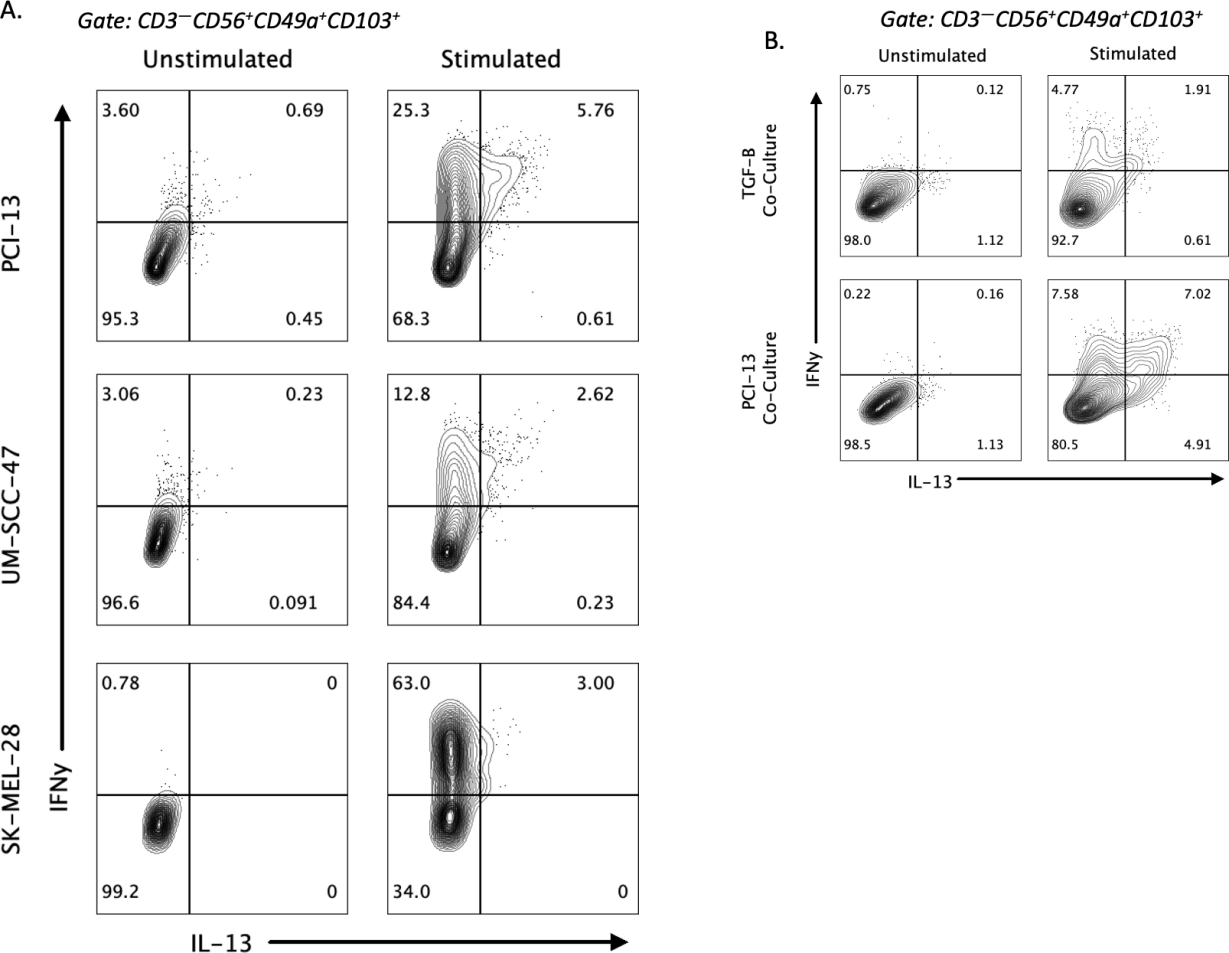
Factors influencing induction of IL-13 in ieILC1-like NK cells. **(A)** Induction of IL-13 expression after stimulation of ieILC1-like NK cells differentiated from peripheral NKs via co-culture with various tumor cell lines. Figure shows representative plot of n=2 donors. **(B)** Induction of IL-13 after co-culture of peripheral NKs with TGF-β and IL-15 to induce ieILC1-like NK cells. Figure shows representative plot of n=3 donors.

We next aimed to uncover possible factors that contribute to the induction of IL-13 production in stimulated ieILC1-like NK cells. First, we assessed if the co-culture process used to differentiate cNKs into ieILC1-like NK cells could influence IL-13 induction. Peripheral NK cells were co-cultured with irradiated tumor cell lines, including UM-SCC-47 HNSCC, K562 chronic myelogenous leukemia, and SK-MEL-28 melanoma. CD49a^+^CD103^+^ ieILC1-like NK cells from these cultures were then stimulated with K562 cells to evaluate IL-13 production. UM-SCC-47 and SK-MEL-28 co-cultures could induce IL-13 production in stimulated ieILC1-like NK cells (**Figure 3A**; **Supplementary Figure 3A**). Thus, there was variation in how cell lines could induce IL-13 by ieILC1-like NK cells.

Given that co-culture of TGF-β and IL-15 could induce CD49a^+^CD103^+^ cells with an inhibitory phenotype and that ieILC1s are TGF-β imprinted, we theorized that TGF-β may be involved in the IL-13 phenotype observed (22, 24). We did not observe any significant difference in our RNAseq data in expression of TGFBR1 and TGFBR2 between IL-13^+^ and IL-13^–^ cells. We performed a co-culture of peripheral blood NK cells with TGF-β and IL-15 and induced a CD49a^+^CD103^+^ phenotype. Stimulation of these cells with K562 showed a IFNγ^+^IL-13^+^ subpopulation that was smaller than the one observed with PCI-13 co-culture (**Figure 3B**). These findings suggest that TGF-β may induce CD49a^+^CD103^+^ NK cells that are capable of IL-13 production upon stimulation, albeit it may not be the only factor capable of doing so. However, a traditional ILC2 stimulating cytokine, IL-25, did not induce IL-13 production (**Supplementary Figure 3B**).

**Figure 4:**
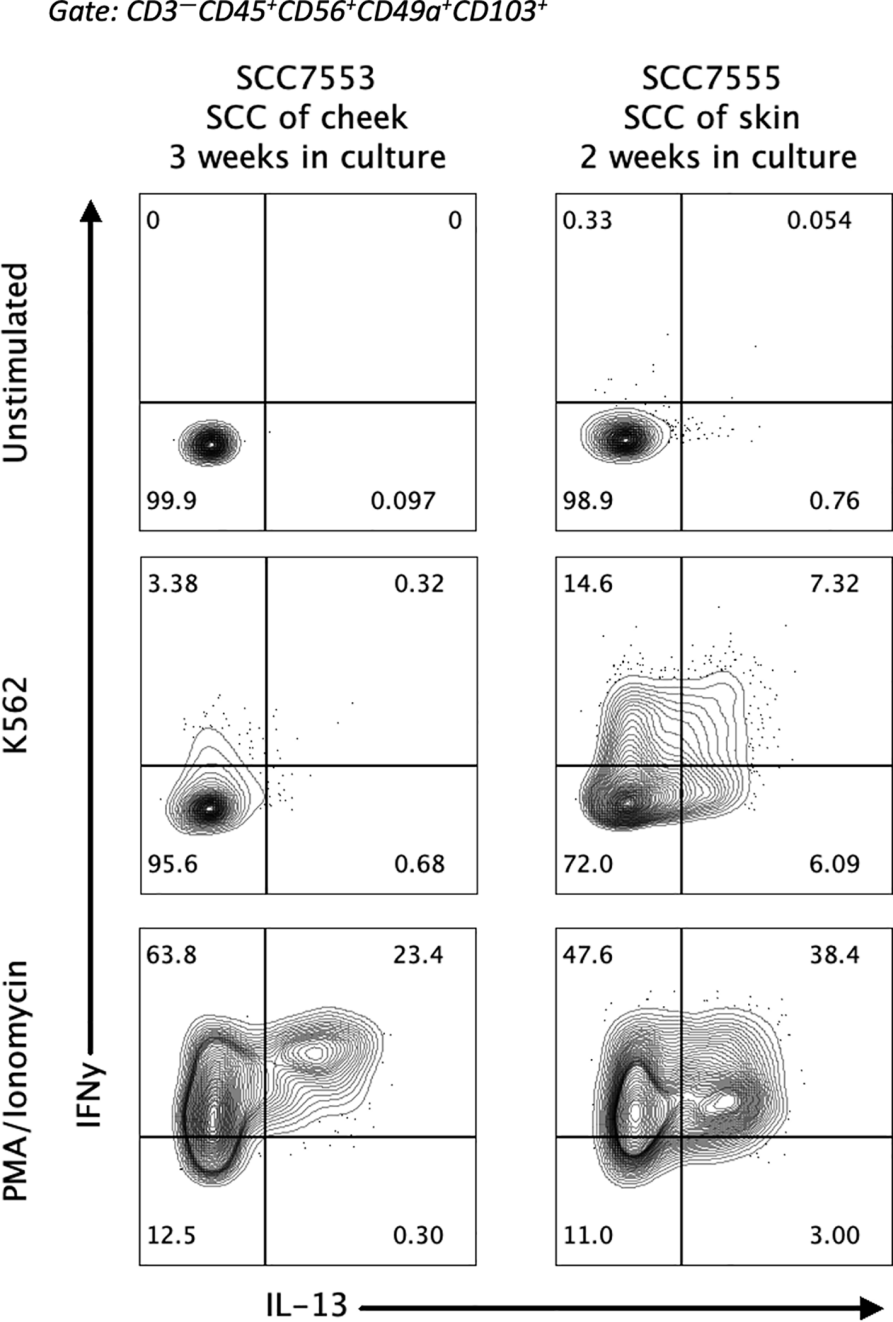
Presence of IL-13^+^ ieILC1-like NK cells in tumor infiltrating lymphocytes. Lymphocytes from patient HNSCC samples were extracted and cultured with IL-2 for the indicated number of weeks. Flow cytometry analysis of IL-13 and IFNγ expression in stimulated ieILC1-like NK cells from these cultures is shown. Figure shows n=2 donors.

To understand if IL-13 producing CD49a^+^CD103^+^ ieILC1-like NK cells have physiologic relevance, we looked in cultures of tumor infiltrating lymphocytes (TIL) from primary tumors for their presence. The relatively small fraction that these cells in the total NK pool made it difficult to observe them in our previous scRNA-seq study of HNSCC (23). Therefore, we isolated and cultured TIL from primary cutaneous SCC and HNSCC tumor samples and stimulated the IL-2 expanded TILs with PMA/ionomycin. Flow cytometric analysis of the stimulated cells revealed a robust subpopulation of IL-13^+^CD49a^+^CD103^+^ NK cells (**Figure 4**). Thus, IL-13 producing CD49a^+^CD103^+^ ieILC1-like NK cells can be identified in stimulated TIL from primary solid tumors, indicating that they may have a physiologic relevance in human disease.

We further attempted to profile this subpopulation’s physiologic relevance by surveying previously published scRNAseq datasets of NK cells. First, we investigated a scRNAseq dataset derived from ILCs from the lamina propria of small intestine from healthy and patients with Crohn’s disease published by Cella et al. and found 24 IL-13^+^ ILCs out of 10,831 ILCs analyzed (**Supplementary Figure 5A**) (31). Next, we investigated a pancancer NK cell scRNAseq dataset published by Tang et al. and similarly only observed 9 IL-13^+^ NK cells out of 11,952 cells analyzed (**Supplementary Figure 5B**) (32). Thus, even before we could subselect for ieILC1s, we find a very small pool of IL-13^+^ cells in these datasets.

To highlight the possible clinical implications of IL-13^+^ ieILC1-like NK cells, we performed a TCGA analysis of survival in HNSCC stratified by IL-13 expression. No statistically significant increase in median overall survival was observed in patients with high IL-13 expression compared to those with low IL-13 expression (**Supplementary Figure 5C**). However, we did find that patients with pathologic stage I tumors had significantly higher IL-13 expression than patients with pathologic stage IV HNSCC (**Supplementary Figure 5D**). Therefore, increased IL-13 expression may be associated with lower pathologic stage.

## 4 Discussion

NK cells and ILCs represent a class of innate immune effectors that have significant heterogeneity in their functional capabilities. Here, we demonstrate cellular heterogeneity within the CD49a^+^CD103^+^ ieILC1-like NK cell subset. We identify a novel population of IL-13^+^ ieILC1-like NK cells that expresses IL-13, a type 2 cytokine, while also expressing canonical type 1 cytokines, like IFNγ, and retaining the transcriptional signature of ILC1s. These data highlight the plasticity of the ieILC1-like NK population, which has not yet been extensively described. While ILC2 plasticity to ILC1 and subsequent reversion to ILC2s has been documented, there is limited data describing populations of NK cells co-expressing type 1 and type 2 cytokines (11, 20, 21). Here, we demonstrate the concurrent production of IFNγ and IL-13 in a subpopulation of ieILC1-like NK cells, pointing to the plasticity of this trNK population. Moreover, we validate that these IL-13^+^ ieILC1-like NK cells still maintain expression of canonical proteins associated with cytotoxicity (IFNγ and CD107a) and proliferative capacity (Ki67).

In addition to IL-13 seen expressed in ieILC1-like NK cells, we also observed increased expression of the CCL20 gene. CCL20 is a chemokine with notable signaling functions in the context of cancer. CCL20, via the CCL20/CCR6 axis, has been documented to promote tumor progression in numerous different types of cancer, including breast, hepatocellular, colorectal, and lung (39–41). CCL20 can promote direct tumor growth, epithelial-mesenchymal transition, angiogenesis, and immunosuppression of CD8^+^ T cells (42). We observe that ieILC1-like NK cells when stimulated have a higher expression of CCL20, which further supports the possibility that ieILC1-like NK cells may have a subset of IL-13^+^ cells that contribute to an immunosuppressive TME.

Furthermore, our data illustrates the heterogeneity within the ieILC1-like NK cell population. Prior work has revealed that the CD49a^+^CD103^+^ subset of NK cells is heterogeneous, with both cytotoxic and inhibitory subsets of cells (23, 24). Our data illustrates heterogeneity from a ILC1/ILC2 plasticity perspective within this population. The signals that predispose a subpopulation of ieILC1-like NK cells to produce IL-13 are not understood. It is also not clear if the IL-13^+^ ieILC1-like NK cells are a distinct functional subset or an intermediate state of ieILC1-like NK cells.

Additionally, we observe that TGF-β may play a role in the induction of IL-13 production in ieILC1-like NK cells. This is consistent with existing data showing that ieILC1s are TGF-β imprinted, and peripheral NK cells cultured with TGF-β and IL-15 can induce an inhibitory phenotype (22, 24). In cancer, TGF-β has been regularly shown to promote cancer progression and can remodel the TME to be immunosuppressive (43). However, we find data that there is no significant upregulation of TGFBR1 or TGFBR2 on DGEA of IL-13^+^ compared to IL-13^–^ cells. Thus, we cannot conclude whether TGF-β is the sole driver of type 2 cytokine production. Further experiments highlighting if TGF-β abrogation are important to clarify the necessity of TGF-β in induction of IL-13; however, ieILC1-like NK cells are TGF-β imprinted and require TGF-β for differentiation of cNKs to ieILC1-like NK cells (23). Thus, parsing whether TGF-β induces IL-13 in ieILC1-like NK cells is a challenge.

Importantly, we were able to observe IL-13^+^ ieILC1-like NK cells among lymphocytes isolated and expanded from the TME of HNSCC patient samples. This indicates that the IL-13^+^ ieILC1-like NK phenotype may have a physiologic role in tumors. However, the functions of these IL-13^+^ ieILC1-like NK cells need to be further elucidated, as IL-13 has been shown to have complex and unclear roles in the TME. Assays to further characterize these cells functionally face the challenge that the IL-13^+^ ieILC1-like NK subset is a small population of cells only identified upon stimulation. Moreover, in our efforts to evaluate these cells in existing scRNAseq datasets, we could not identify a robust population of IL-13^+^ cells. However, given we only observe the IL-13 phenotype after stimulation, the lack of IL-13^+^ cells in published data is not surprising. In future studies, we aim to develop *in vitro* methods of expanding these IL-13^+^ cells so that we can profile their functional capabilities further, rather than relying on markers like CD107a to provide insight into their function.

It remains unclear whether IL-13 has pro-tumorigenic or anti-tumorigenic properties, and it may indeed have both depending on context. In our data, we find that IL-13^+^ ieiLC1-like NK cells express markers of cytotoxicity like CD107a and higher levels of IL-13 in HNSCC were associated with lower pathologic stage, supporting a possible anti-tumor role for this cell subset. However, a direct comparison of cytotoxicity between IL-13^+^ and IL-13^–^ ieILC1-like NK cells remains necessary, and future work will include further *in vitro* and *in vivo* functional assays to characterize this subpopulation.

## Data Availability

The data presented in the study are deposited in the Gene Expression Omnibus repository, accession number GSE289886.
