# Supplementary material for: An intraepithelial ILC1-like natural killer cell subset produces IL-13
Source: Front Immunol. 2025 Mar 6;16:1521086. doi: 10.3389/fimmu.2025.1521086 (PMC11922857; doi:10.3389/fimmu.2025.1521086)
Supplement: Supplementary file 1 [file DataSheet1.docx]

Supplementary Material

## Supplementary Figures

**
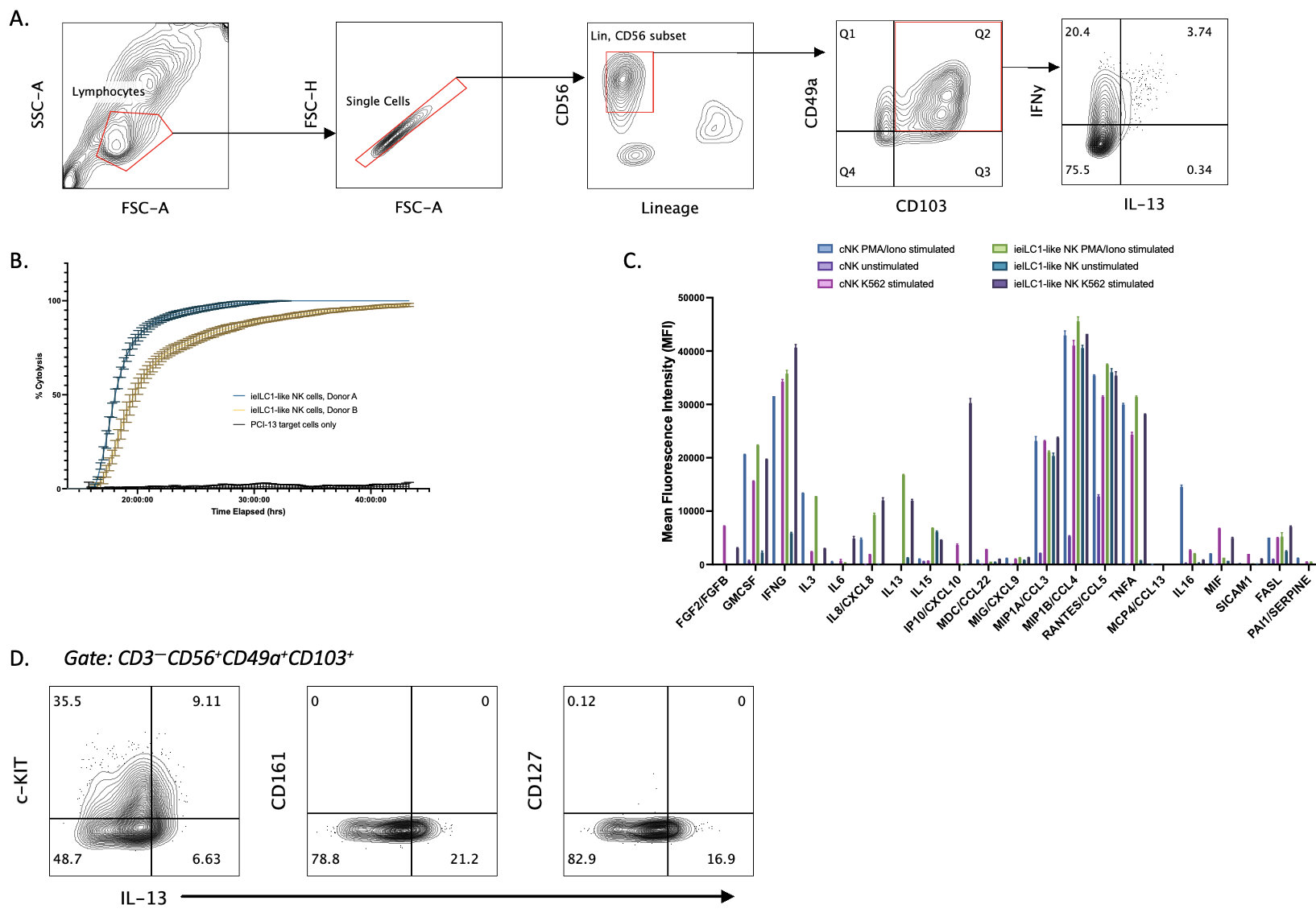
**

**Supplementary Figure 1.** Stimulation of ieILC1-like NK cells. A) Gating schema for ieILC1-like NK cells. After gating on singlet lymphocytes, we analyzed cells that were CD3–CD14–CD19–CD20– and CD56+CD49a+ for this analysis involving cNKs. ieILC1-like NK cells are typically characterized as CD49a+CD103+. B) xCelligence cytotoxicity assay showing ieILC1-like NK cells differentiated from peripheral NK cells were cytotoxic against PCI-13 target cells. Figure shows a representative plot for 2 donors analyzed. C) Comprehensive results of Luminex immunoassay showing cytokines produced by cNKs and ieILC1-like NK cells with and without stimulation. D) Expression of ILC2—associated markers assayed via flow cytometry.


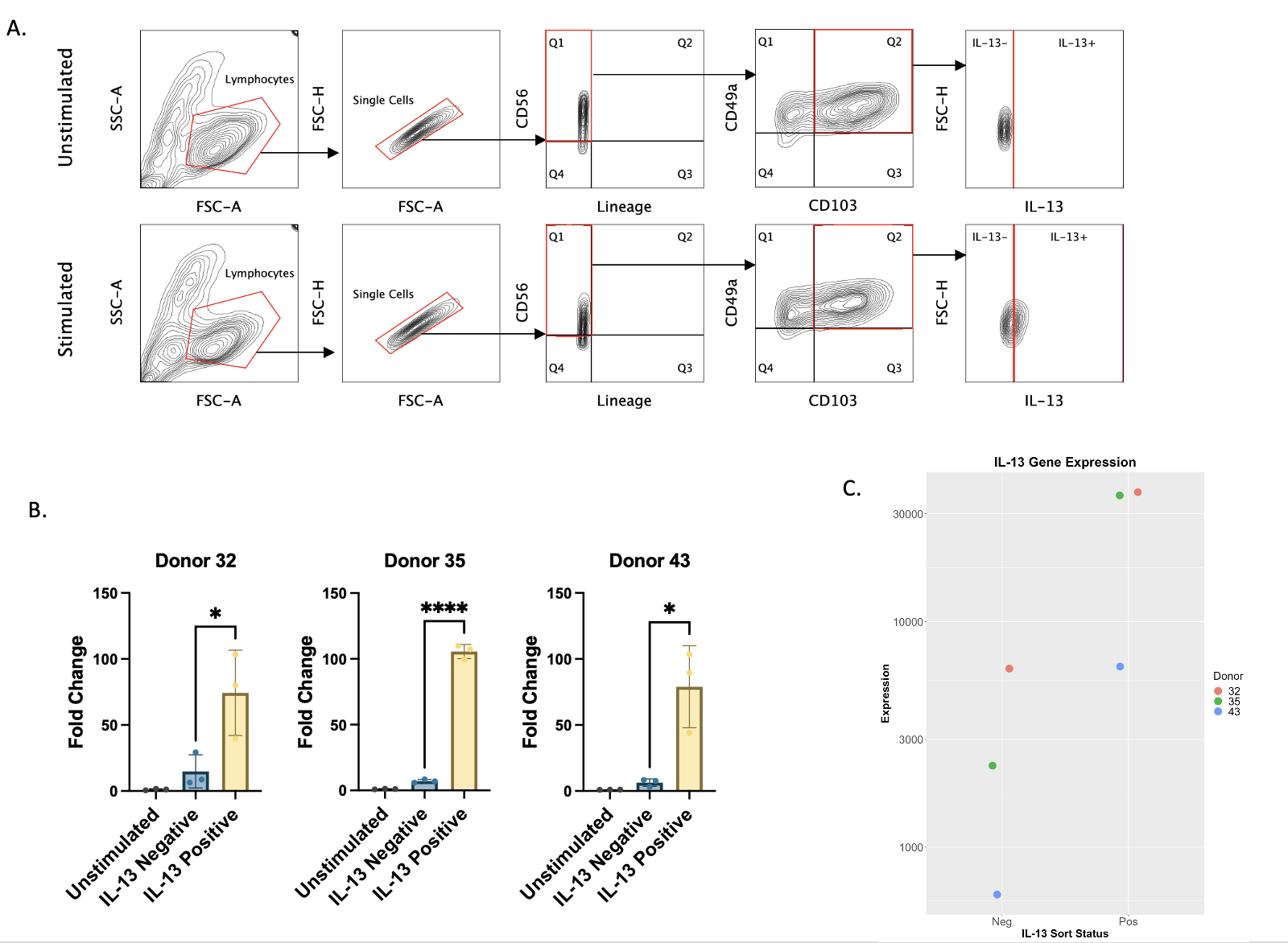


**Supplementary Figure 2**. Sorting of IL-13+ ieILC1-like NK cells and RNA sequencing. A) Gating schema for IL-13+ ieILC1-like NK cell sorting. We analyzed CD3–CD14–CD19–CD20– and CD56+CD49a+CD103+ cells, with IL-13 expression measured by a cytokine secretion assay. B) Post-sort quality analysis of IL13 gene expression in IL-13+ and IL-13– sorted populations via qRT-PCR. C) Post-RNA sequencing analysis of IL13 expression levels in IL-13– and IL-13+ groups across all donors. *p<0.05, ****p<0.0001


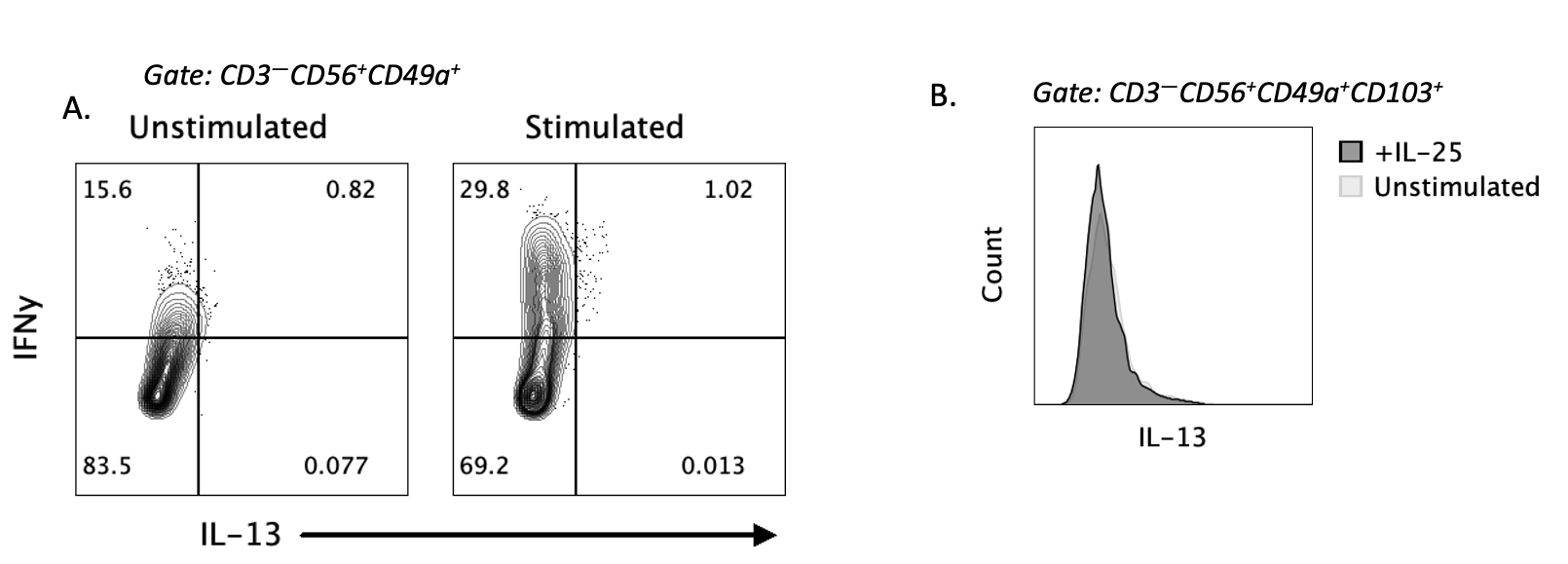


**Supplementary Figure 3.** Induction of IL-13+ ieILC1-like NK cells. A) Induction of IL-13 expression after stimulation of CD49a+ NK cells differentiated from peripheral NKs via co-culture with K562, gated on CD49a+ NK cells instead of CD49a+CD103+ NK cells. B) Flow cytometry analysis of IL-13 production after stimulation of ieILC1-like NK cells with IL-25.


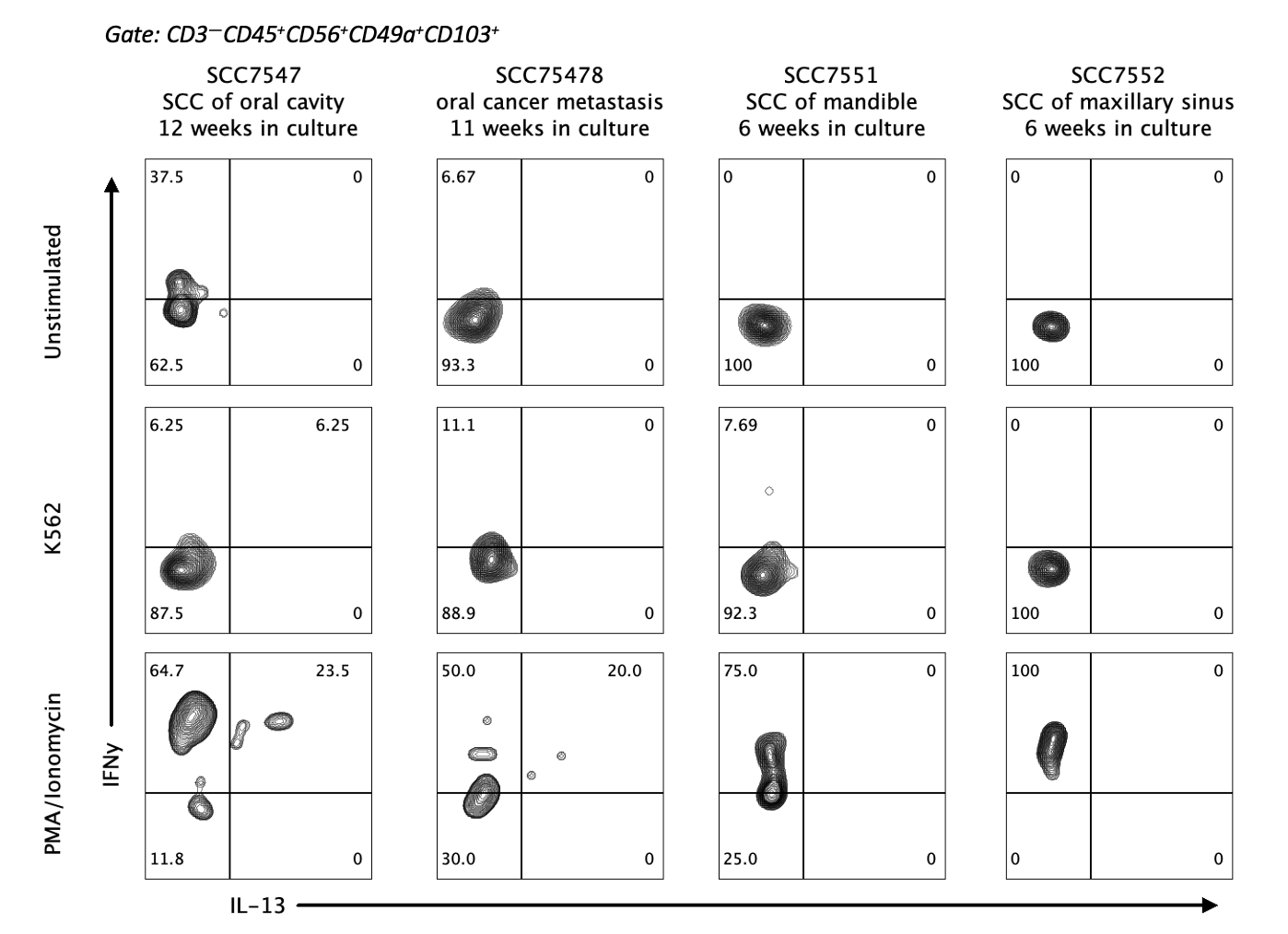


**Supplementary Figure 4.** In vivo identification and effects of ieILC1-like NK cells producing IL-13. Lymphocytes from patient HNSCC samples were extracted and cultured with IL-2 for the indicated number of weeks. Flow cytometry analysis of IL-13 and IFNy expression in stimulated ieILC1-like NK cells from these cultures is shown for all samples in which no IL-13+ ieILC1-like NK cells were shown.


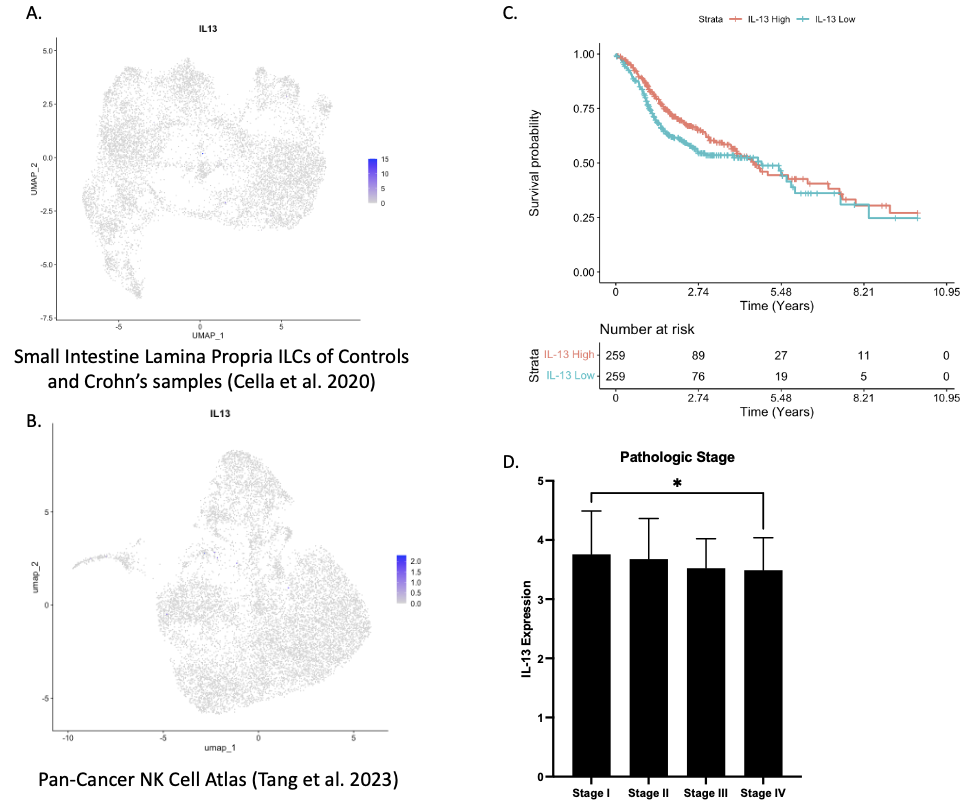


**Supplementary Figure 5.** Physiologic and clinical significance of IL-13+ ieiLC1-like NK cells. A-B) Analysis of previously published scRNAseq datasets. C) Kaplan-Meier curve of overall survival stratified by median IL-13 expression in TCGA HNSCC patients. D) Average IL-13 expression stratified by pathologic tumor stage in TCGA HNSCC patients. *p<0.05
